# Effects of Dietary Supplementation with Chitosan on the Muscle Composition, Digestion, Lipid Metabolism, and Stress Resistance of Juvenile Tilapia (*Oreochromis niloticus*) Exposed to Cadmium-Induced Stress

**DOI:** 10.3390/ani14040541

**Published:** 2024-02-06

**Authors:** Qin Zhang, Yi Xie, Yuanhui Zhang, Enhao Huang, Liuqing Meng, Yongqiang Liu, Tong Tong

**Affiliations:** 1 School of Marine Sciences and Biotechnology, Guangxi Minzu University, Nanning 530008, China; zhangqin@gxmzu.edu.cn (Q.Z.); xy463317779@163.com (Y.X.); zyh_sophie@163.com (Y.Z.); 15113161786@163.com (E.H.); mliuqing0713@126.com (L.M.); 2 Guangxi Key Laboratory for Polysaccharide Materials and Modifications, Nanning 530008, China; 3 Guangxi Marine Microbial Resources Industrialization Engineering Technology Research Center, Nanning 530008, China

**Keywords:** chitosan, cadmium, tilapia, stress resistance, lipid metabolism

## Abstract

**Simple Summary:**

Long-term exposure to cadmium can cause liver toxicity, kidney toxicity, bone toxicity, and cancer in aquatic animals, which seriously endangers the health of aquatic animals and the quality of aquatic products. Chitosan can be combined with cadmium in aquaculture water through adsorption, thus alleviating the toxic effects of cadmium on aquatic animals. In this study, juvenile tilapia was fed with a formulated feed containing five different levels (0%, 0.5%, 1.0%, 1.5%, and 2.0%) of chitosan for 60 days, while the water in all experimental groups contained a Cd^2+^ concentration of 0.2 mg/L. The results indicated that dietary chitosan supplementation could alleviate the effects of Cd^2+^ stress on the muscle composition, digestive enzymes, lipid metabolism, and stress resistance, and their related gene expression, of juvenile tilapia.

**Abstract:**

The aim of this study was to investigate the effects of dietary chitosan supplementation on the muscle composition, digestion, lipid metabolism, and stress resistance, and their related gene expression, of juvenile tilapia (*Oreochromis niloticus*) subjected to cadmium (Cd^2+^) stress. Juvenile tilapia with an initial body weight of 21.21 ± 0.24 g were fed with a formulated feed containing five different levels (0%, 0.5%, 1.0%, 1.5%, and 2.0%) of chitosan for 60 days, while the water in all experimental groups contained a Cd^2+^ concentration of 0.2 mg/L. The results showed that, compared with the control group (0% chitosan), the contents of crude fat and crude protein in the muscle, the activities of lipase, trypsin, and amylase in the intestine, as well as the relative expression levels of metallothionein (*mt*), cytochrome P450 1A (*cyp1a*), carnitine palmitoyltransferase-1 (*cpt-1*), peroxisome proliferator-activated receptor alpha (*pparα*), peroxisome proliferator-activated receptor gamma (*pparγ*), hormone-sensitive lipase (*hsl*), lipoprotein lipase (*lpl*), malate dehydrogenase (*mdh*), leptin (*lep*), fatty acid synthase (*fas*), sterol regulatory element-binding protein 1 (*srebp1*), and stearoyl-CoA desaturase (*scd*) genes in the liver of juveniles were significantly increased (*p* < 0.05). In conclusion, dietary chitosan supplementation could alleviate the effects of Cd^2+^ stress on the muscle composition, digestive enzymes, lipid metabolism, and stress resistance, and their related gene expression, of juvenile tilapia, and to some extent reduce the toxic effect of Cd^2+^ stress on tilapia.

## 1. Introduction

The element cadmium is one of the most toxic heavy-metal pollutants in the environment which can enter the aquatic ecosystem through industrial wastewater. Cadmium can pass through the food chain and accumulate within organisms at higher trophic levels, thereby inducing changes in biological physiology and affecting biological behavior [1]. The entry and accumulation of cadmium in organisms generally occur through respiration, skin absorption, and dietary intake, leading to toxic effects on the organisms [2]. Long-term exposure to cadmium can result in liver, kidney and skeletal toxicity and carcinogenicity in aquatic animals, posing a significant threat to their health and the quality of aquatic products [3]. The concentration of cadmium in several rivers in China generally ranged from 10 to 500 ng/L, with levels exceeding 1 mg/L reported in certain industrialized areas [4]. In accordance with the guidelines set by the US Environmental Protection Agency (USEPA), a permissible limit of 0.005 mg/L for cadmium (Cd(II)) in the water has been established [5]. At present, methods of removing cadmium in water pollution treatment are mainly physical and chemical methods and biological methods [6].

The utilization of chitosan for mitigating the deleterious impact of heavy-metal contamination on aquaculture organisms represents a prevalent treatment approach for the elimination of heavy-metal pollution in water [7]. An adsorption capacity of 10.0 mg/g for cadmium by chitosan beads has been reported (at pH 6–7) [8]. The environmentally friendly and sustainable nature of chitosan has garnered significant attention in the fields of water treatment and environmental protection. It is widely acknowledged that chitosan exhibits exceptional adsorption capabilities for heavy metals, thereby presenting a wide range of potential applications [9]. The results of various studies have demonstrated that low-molecular-weight chitosan has the capacity to adsorb cadmium in aquaculture water, thereby alleviating its toxic impact on aquatic organisms [10]. At the same time, the utilization of chitosan as a novel adsorbent demonstrates its exceptional affinity toward lead and copper ions in aqueous solutions, thereby showcasing remarkable adsorption capacity [11,12]. Additionally, chitosan exhibits the ability to adsorb cadmium ions in aqueous environments and acts as a natural marine prebiotic for enhancing the immune response and disease resistance of aquatic organisms, suppressing the proliferation of harmful microorganisms and accelerating wound healing. Its biodegradability and unique molecular structure are widely recognized within the aquaculture industry [10,11]. However, the long-term inclusion of chitosan in animal feed has been observed to induce weight reduction in fish. This phenomenon can be attributed to the intricate interplay between chitosan and the lipids, cholesterol, fat-soluble vitamins, and minerals present in fish feed, resulting in diminished nutrient absorption and subsequent loss of body mass [13]. Therefore, it is imperative to determine the optimal dosage of chitosan in fish feed.

The genetically improved farmed tilapia (GIFT, *Oreochromis niloticus*) is a genetically modified strain of tilapia that exhibits desirable traits, including exceptional salt tolerance, rapid growth rate, high meat yield, robust cold resistance, ease of domestication, and superior catchability, which have gained significant favor among both farmers and consumers [14,15]. Currently, research on tilapia primarily focuses on the physiological toxicity of heavy-metal pollution to fish. However, there is a limited number of studies investigating the potential mitigation of heavy-metal stress through chitosan supplementation in tilapia feed. The objective of this study was to investigate the effects of dietary chitosan supplementation on the muscle composition, digestive enzymes, lipid metabolism, and stress resistance, and their related gene expression, of juvenile tilapia under cadmium exposure. This study aims to provide scientific insights into the application of chitosan in aquaculture practices and the management of heavy-metal pollution in aquatic environments.

## 2. Materials and Methods

### 2.1. Experimental Materials

The cadmium chloride (CdCl_2_) utilized in this study was of analytical purity and was procured from Sigma-Aldrich (Shanghai) Trading Co., Ltd., Shanghai, China. The chitosan used was food-grade with a deacetylation degree ≥ 95% and viscosity ranging from 100–200 mpa.s, procured from Shanghai Yichen Biotechnology Co., Ltd., Shanghai, China. The origin of chitosan was the marine crab shell. The chitosan had a particle size smaller than 74 μm (200 mesh sieve). All the feed materials were procured from Nanning Tongwei Feed Co., Ltd., Nanning, China, and they were animal food grade.

### 2.2. Experimental Diets

Based on prior research findings [10,14], five iso-nitrogen, iso-lipid, and iso-energy feeds were used in this study. The control diet was designated as the 0% group without chitosan supplementation and the remaining experimental diets were supplemented with chitosan at concentrations of 0.5%, 1.0%, 1.5%, and 2.0%, respectively designated as the 0.5% group, the 1.0% group, the 1.5% group and the 2.0% group. The composition of the experimental diets is shown in Table 1.

Different diets were made separately into pellets as follows. First, precise weighing of all raw materials based on the prescribed formula (Table 1). Second, except for fish oil and soybean oil, all solid ingredients underwent meticulous pulverization followed by passage through a 60-mesh sieve. They were then introduced into mixing equipment where they underwent comprehensive blending for a duration of 15 min. Third, the mixture was tempered and matured by a temperer, extruded by an expanding machine to make particles with a particle size of 1–1.5 mm, and then dried in a blast-drying oven until the water content was below 100 g/kg. Finally, the remaining fish oil and soybean oil were evenly distributed onto the feed particles through the employment of a vacuum-spraying technique. The finished feed was placed separately in a marked plastic bag and stored at −20 °C.

### 2.3. Experimental Fish, Acclimatization, and Culture

The juvenile GIFT used in this experiment was approved by the Ethics Committee of Guangxi Minzu University, Nanning, China (No. GXMZU-2022-001).

A total of 600 juvenile GIFT were sourced from the Guangxi Academy of Fishery Sciences’ seed-breeding farm, Nanning, China. After a 15 min disinfection period using a potassium permanganate solution with a concentration of 20 mg/L, the juvenile GIFT were subsequently acclimated in the laboratory aquaculture system of Guangxi Minzu University for a duration of 14 days. The acclimatization conditions were water temperature 26 ± 1 °C, pH 7–8, dissolved oxygen > 9 mg/L, and a light–dark period of 12 h: 12 h. The juvenile GIFT in each group were fed three times a day at 9:00, 14:00, and 19:00 using a control diet (0% chitosan), and the daily feeding quantity was determined by feeding until the fish no longer exhibited feeding behavior at the feeding time.

After a 14 day acclimatization period, a total of 450 juvenile GIFT with an initial body weight of 21.21 ± 0.24 g were randomly selected and assigned to five groups with three replicates per group, making a total of 15 tanks with 30 fish in each tank for the formal experiment. The total volume of each tank is 1000 L, and the volume of the aquaculture water is 720 L. The juvenile GIFT were reared in the same breeding conditions and acclimatization conditions mentioned above.

According to the previous experimental findings of this study, when the concentration of Cd^2+^ in water exceeded 0.2 mg/L, there was a significant impact on the growth performance of juvenile GIFT. Therefore, a standardized concentration of Cd^2+^ at 0.2 mg/L was maintained in each aquaculture water sample (a total of 15 tanks).

During the whole experiment period, meticulous observations were conducted on the juvenile GIFT in each group to evaluate their condition and document activities such as feeding and body coloration. In the case of juvenile GIFT mortality, immediate removal and documentation procedures were implemented. A daily replacement of one-third of the aquaculture water was performed in each tank, accompanied by the addition of Cd^2+^ venom at an equivalent volume and concentration. Throughout the 60 day duration of the experiment, a relatively constant Cd^2+^ concentration at 0.2 mg/L was maintained in all tanks.

### 2.4. Sampling

Following a 60 day culture period, the juvenile GIFT were subjected to a 24 h fasting period. Subsequently, 18 fish were randomly selected from each experimental group (6 fish per tank). The sample fish were individually anesthetized using 200 mg/L ethyl 3-aminobenzoate mesylate (MS-222, Adamas, Shanghai Adamas Reagent Co., Ltd., Shanghai, China), and their muscles, intestines, and livers were taken and placed in labeled sample bags and stored in liquid nitrogen. After all the samples were sampled, they were preserved at −80 °C in an ultra-low-temperature refrigerator for subsequent analysis.

### 2.5. Determination of Digestive Enzyme Activity

The activities of α-amylase, lipase, and trypsin in the intestines of juvenile GIFT were determined by the microplate method using an ELISA analyzer (RT-6100, Rayto, Shenzhen, China) and assay kits. The assay kits were made by Nanjing Jiancheng Bioengineering Institute (Nanjing, China), and all the instruction manuals can be found and downloaded at http://www.njjcbio.com/ (accessed on 1 January 2024).

The determination of α-amylase was performed by starch–iodine colorimetry method. α-amylase unit definition is a s follows: Each milligram of protein in the tissue underwent substrate interaction at a temperature of 37 °C for a duration of 30 min, and the hydrolysis of 10 mg starch was designated as one unit of α-amylase activity (U/mgprot).

The determination of α-amylase was made by the colorimetric method. Lipase unit definition was conducted as follows. The reaction system operated at a temperature of 37 °C, where each gram of protein in the tissue underwent substrate reaction for a duration of 1 min. The utilization of 1 μmol of substrate corresponds to one unit of lipase activity (U/mgprot).

The determination of trypsin was made by the ultraviolet colorimetry method. Trypsin unit definition was performed as follows: the trypsin present in each milligram of protein in the tissue caused a change in absorbance of 0.003 per minute in a pH of 8.0 and 37 °C environment, exhibiting one unit of trypsin activity (U/mgprot).

### 2.6. Determination of Muscle Composition

Muscle crude protein was determined by the Kjeldahl nitrogen determination method (GB/T 6432-2018, Chinese standards) [15]. Muscle crude fat was determined by the Soxhlet extraction method (GB/T 6433-2006, Chinese standards) [16]. Muscle moisture was determined by the oven-drying constant weight method at 105 °C (GB/T 6435-2014, Chinese standards) [17]. Muscle ash was obtained by the burning method in a muffle furnace at 550 °C (GB/T 6438-2007, Chinese standards) [18].

### 2.7. Determination of Gene Expression

The expression levels of sonic hedgehog (*shh*), heat shock protein (*hsp*), metallothionein (*mt*), cytochrome P450 1A (*cyp1a*), carnitine palmitoyltransferase-1 (*cpt-1*), peroxisome proliferator-activated receptor alpha (*pparα*), hormone-sensitive lipase (*hsl*), lipoprotein lipase (*lpl*), malate dehydrogenase (*mdh*), leptin (*lep*), peroxisome proliferator-activated receptor gamma (*pparγ*), fatty acid synthase (*fas*), sterol regulatory element-binding protein 1 (*srebp1*), squalene epoxidase (*sqle*), and stearoyl-CoA desaturase (*scd*) genes in the liver of juvenile GIFT were determined using real-time quantitative polymerase chain reactions (RT-qPCRs). *β-actin* was employed as the internal reference gene. Forward and reverse primers were designed based on the mRNA sequences of tilapia available in the National Center for Biotechnology Information (NCBI) database, and synthesized by Shanghai Sangon Bioengineering Technology Service Co., Ltd., Shanghai, China. Detailed primer information is provided in Table 2.

The RT-qPCR method of Liu et al. was applied [19], and the brief steps were as follows. First, the total RNA was extracted from the liver of juvenile GIFT using the Steady Pure Universal RNA Extraction Kit produced by Accurate Biology Biotechnology Engineering Ltd., Changsha, China. The instructions provided in the kit offer additional information. The RNA quantity and purity were determined using spectrophotometry, specifically by assessing the absorbance ratio of 260:280 nm with an ND-2000 spectrophotometer (Thermo, Waltham, MA, USA). The integrity of the RNA was evaluated through gel electrophoresis on a 1% (*w*/*v*) agarose TAE gel stained with Gel RedTM nucleic acid stain (UVP, Upland, CA, USA). Second, 1 μL of total RNA was reverse transcribed into cDNA using the Evo M-MLV reverse transcription kit (Accurate Biology Biotechnology Engineering Ltd., Changsha, China). The reverse transcription process consisted of incubation at 30 °C for 10 min, followed by 42 °C for 15 min, and a final step at 95 °C for 5 min. After cooling to 5 °C for an additional 5 min, the reaction mixture containing 2 × Taqman PCR mix (25 μL), upstream primer (10 μM) (2.5 μL), downstream primer (10 μM) (2.5 μL), DNA templates (1 μL), and ddH_2_O (19 μL) was prepared in a total volume of 50 μL per reaction cycle as specified by the manufacturer’s instructions provided with the kit from Accurate Biology Biotechnology Engineering Ltd., Changsha, China. After the PCR reaction, the cDNA sample was obtained. Third, the RT-qPCR assays were conducted using a LightCycler 96 RT-qPCR Detection System (Roche, Basel, Switzerland) and the SYBR Green Pro Taq HS-qPCR kit produced by Accurate Biology Biotechnology Engineering Ltd., Changsha, China. The RT-qPCR reactions were carried out with 2 μL of gDNA Clean Reaction Mix Ver.2, 4 μL of 5 × Evo M-MLVRT Reaction Mix Ver.2, 1 μL of Forward primer, 1 μL of Reverse primer, 2 μL of cDNA, and 10 μL of RNase free water, in a total reaction volume of 20 μL. The thermal cycling conditions for the RT-qPCR reactions included an initial denaturation step at 95 °C for 10 s followed by amplification with 40 cycles consisting of denaturation at 95 °C for 60 s; annealing at 60 °C for 30 s; and extension at 72 °C for 90 s. The specificity of the reactions was confirmed by analyzing the melting curve obtained during heating to 95 °C.

The 2^−∆∆Ct^ method [20] was applied to calculate the relative expression levels of *shh*, *hsp*, *mt*, *cyp1a*, *cpt-1*, *pparα*, *hsl*, *lpl*, *mdh*, *lep*, *pparγ*, *fas*, *srebp1*, *sqle*, and *scd* genes.

### 2.8. Data Calculation and Statistics

The data underwent initial processing in Microsoft Excel 2023 (Version number: 16.78 [23100802], Microsoft Corporation, Washington, WA, USA). Subsequently, a one-way analysis of variance (ANOVA) was performed using the IBM SPSS 26 software package (International Business Machines Corporation, Armonk, NY, USA). Normality and homogeneity of variances among the groups were evaluated before conducting the statistical tests. For comparisons between groups, Duncan’s multiple-range test was utilized. Statistical significance was set at *p* < 0.05 level and results are reported as mean ± standard error (mean ± SE).

## 3. Results

### 3.1. Effects of Dietary Supplementation with Chitosan on the Muscle Composition of Juvenile GIFT Exposed to Cadmium-Induced Stress

Compared to the control group (0% chitosan), dietary supplementation with chitosan significantly increased the contents of crude protein and crude fat in the muscle of juvenile GIFT exposed to cadmium-induced stress (*p* < 0.05). The 1.5% group exhibited the highest contents of crude protein and crude lipid, and there were no significant differences compared to the 0.5%, 1.0%, and 2.0% groups (*p* > 0.05), respectively. However, there were no significant differences observed in the moisture and ash content in their muscles (*p* > 0.05), as shown in Table 3.

### 3.2. Effects of Dietary Supplementation with Chitosan on the Intestinal Digestive Enzyme Activities of Juvenile GIFT Exposed to Cadmium-Induced Stress

Compared to the control group (0% chitosan), dietary supplementation with chitosan significantly increased the activities of lipase, trypsin, and α-amylase in the intestine of juvenile GIFT exposed to cadmium-induced stress (*p* < 0.05). The 1.5% group exhibited the highest activities of lipase and trypsin, and there were no significant differences compared to the 0.5%, 1.0%, and 2.0% groups (*p* > 0.05). The 2.0% group exhibited the highest activity of α-amylase, and there were no significant differences compared to the 0.5%, 1.0%, and 1.5% groups (*p* > 0.05), as shown in Table 4.

### 3.3. Effects of Dietary Supplementation with Chitosan on the Relative Expression Levels of Stress-Resistance Genes in the Liver of Juvenile GIFT Exposed to Cadmium-Induced Stress

**Figure 1:**
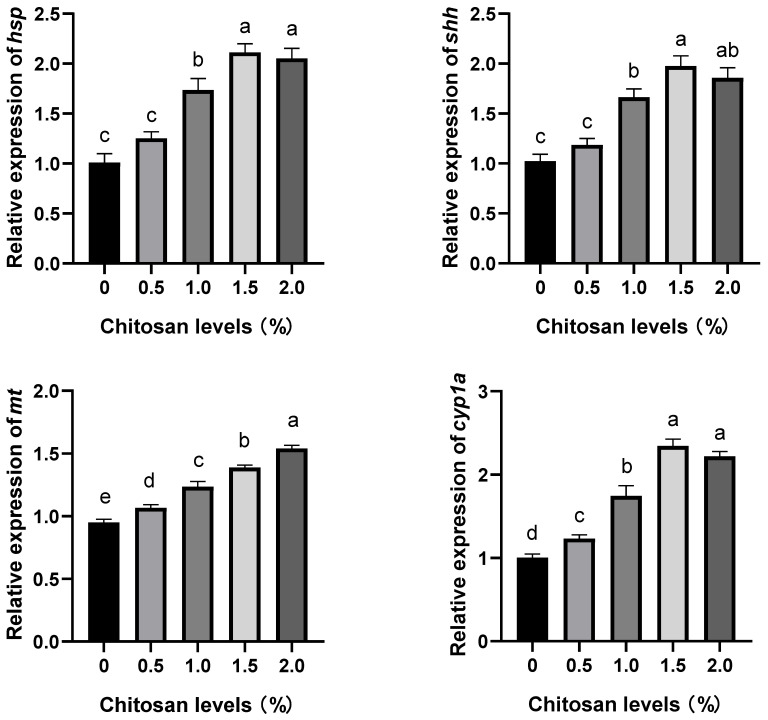
Effects of dietary supplementation with chitosan on the relative expression levels of stress-resistance genes in the liver of juvenile GIFT exposed to cadmium-induced stress, where *hsp* indicates heat shock protein, *shh* indicates sonic hedgehog, *mt* indicates metallothionein, and *cyp1a* indicates cytochrome P450 1A. All above data are mean ± SE (*n* = 3 × 3 × 3), which means 3 parallel groups for each chitosan level, 3 fish samples for each parallel group, and 3 measured times for each fish sample. Different superscript letters in the same figure indicate significant differences among the data (*p* < 0.05).

Compared to the control group (0% chitosan), dietary supplementation with chitosan at concentrations of 1.0%, 1.5%, and 2.0% significantly up-regulated the relative expression levels of the heat shock protein (*hsp*) and sonic hedgehog (*shh*) genes in the liver of juvenile GIFT exposed to cadmium-induced stress (*p* < 0.05). The 1.5% group exhibited the highest relative expression levels of *hsp* and *shh* genes, which were significantly higher than those in the 1.0% group (*p* < 0.05), but no significant differences were observed compared to the 2.0% group (*p* > 0.05). However, the relative expression levels of the *hsp* and *shh* genes in the 0.5% group did not exhibit significant differences compared to those in the control group (*p* > 0.05), as shown in Figure 1.

Compared to the control group (0% chitosan), dietary supplementation with chitosan at concentrations of 0.5%, 1.0%, 1.5%, and 2.0% significantly up-regulated the relative expression level of the metallothionein (*mt*) gene in the liver of juvenile GIFT exposed to cadmium-induced stress (*p* < 0.05). The 2.0% group exhibited the highest relative expression level of the *mt* gene, which was significantly higher than that in the 0.5%, 1.0%, and 1.5% groups (*p* < 0.05), as shown in Figure 1.

Compared to the control group (0% chitosan), dietary supplementation with chitosan at concentrations of 0.5%, 1.0%, 1.5%, and 2.0% significantly up-regulated the relative expression level of the cytochrome P450 1A (*cyp1a*) gene in the liver of juvenile GIFT exposed to cadmium-induced stress (*p* < 0.05). The 1.5% group exhibited the highest relative expression level of the *cyp1a* gene, which was significantly higher than those in the 0.5% and 1.0% groups (*p* < 0.05), but no significant difference was observed compared to the 2.0% group (*p* > 0.05), as shown in Figure 1.

### 3.4. Effects of Dietary Supplementation with Chitosan on the Relative Expression Levels of Lipid Metabolism Genes in the Liver of Juvenile GIFT Exposed to Cadmium-Induced Stress

**Figure 2:**
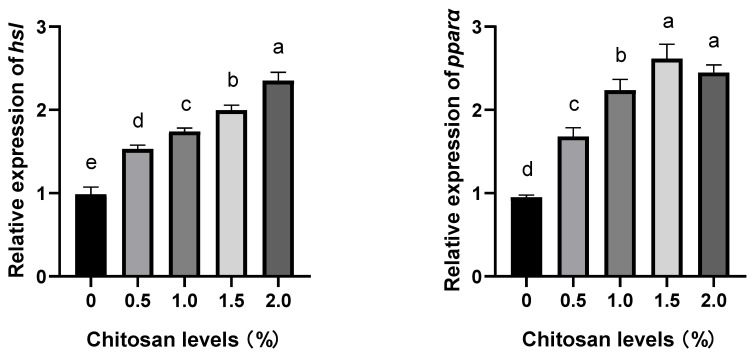

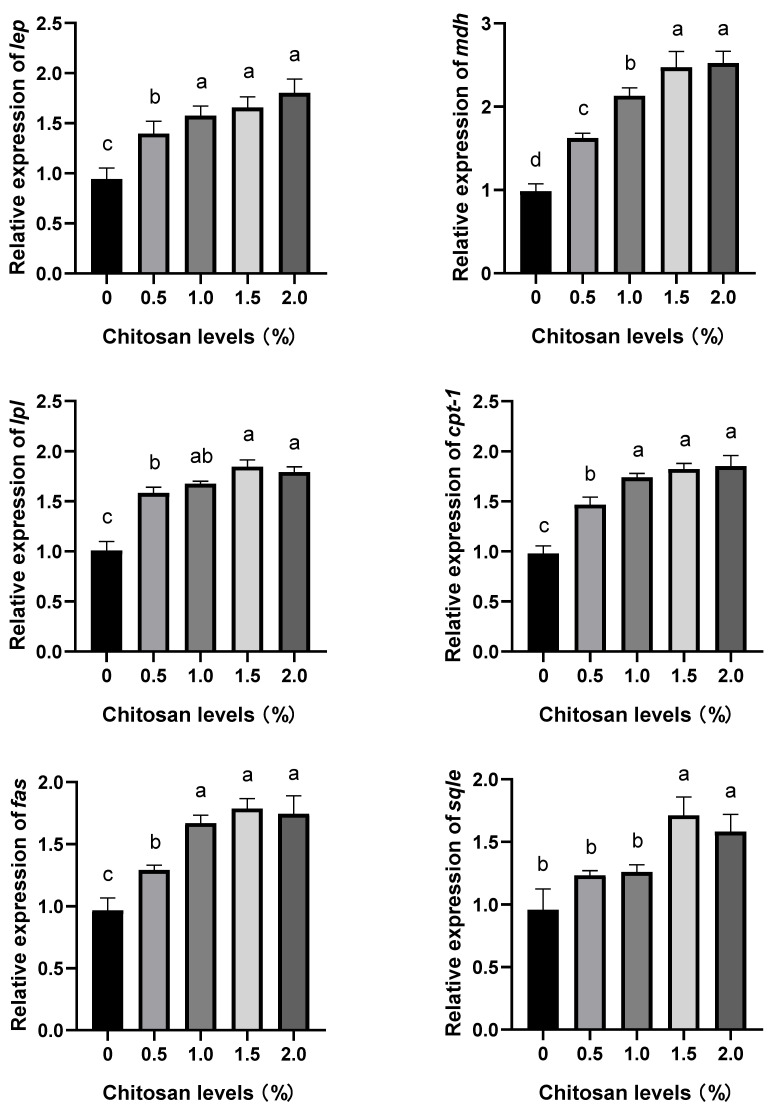

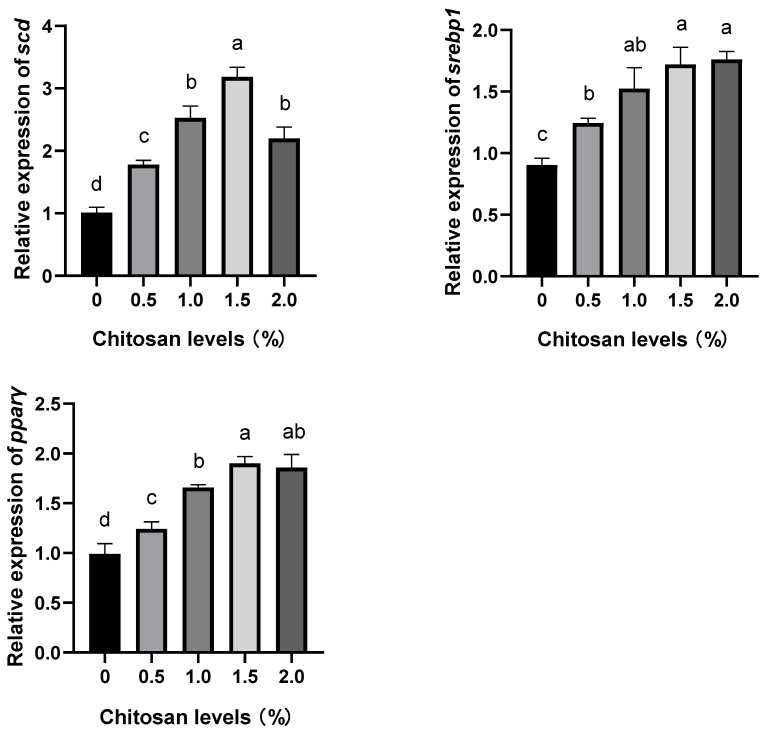
Effects of dietary supplementation with chitosan on the relative expression levels of lipid metabolism genes in the liver of juvenile GIFT exposed to cadmium-induced stress, where *hsl* indicates hormone-sensitive lipase, *pparα* indicates peroxisome proliferator-activated receptor alpha, *lpl* indicates lipoprotein lipase, *mdh* indicates malate dehydrogenase, *cpt-1* indicates carnitine palmitoyltransferase 1, *lep* indicates leptin, *fas* indicates fatty acid synthase, *scd* indicates stearoyl-CoA desaturase, *sqle* indicates squalene epoxidase, *pparγ* indicates peroxisome proliferator-activated receptor gamma, and *srebp1* indicates sterol regulatory element-binding protein 1. All above data are mean ± SE (*n* = 3 × 3 × 3), which means 3 parallel groups for each chitosan level, 3 fish samples for each parallel group, and 3 measured times for each fish sample. Different superscript letters in the same figure indicate significant differences among the data (*p* < 0.05).

Compared to the control group (0% chitosan), dietary supplementation with chitosan at concentrations of 0.5%, 1.0%, 1.5%, and 2.0% significantly up-regulated the relative expression levels of hormone-sensitive lipase (*hsl*), peroxisome proliferator-activated receptor alpha (*pparα*), lipoprotein lipase (*lpl*), malate dehydrogenase (*mdh*), carnitine palmitoyltransferase 1 (*cpt-1*), leptin (*lep*), fatty acid synthase (*fas*), stearoyl-CoA desaturase (*scd*), peroxisome proliferator-activated receptor gamma (*pparγ*)*,* and sterol regulatory element-binding protein 1 (*srebp1*) genes in the liver of juvenile GIFT exposed to cadmium-induced stress (*p* < 0.05). The 2.0% group exhibited the highest relative expression level of the *hsl* gene, which was significantly higher than that of the 0.5%, 1.0%, and 1.5% groups (*p* < 0.05). The 1.5% group exhibited the highest relative expression levels of *pparα* and *pparγ* genes, which were significantly higher than those in the 0.5% and 1.0% groups (*p* < 0.05), but no significant differences were observed in the 2.0% group (*p* > 0.05). The 1.5% group exhibited the highest relative expression levels of *lpl* and *fas* genes, which were significantly higher than those in the 0.5% group (*p* < 0.05), but no significant differences were observed in the 1.0% and 2.0% groups (*p* > 0.05). The 2.0% group exhibited the highest relative expression levels of the *mdh* gene, which was significantly higher than those in the 0.5% and 1.0% groups (*p* < 0.05), but no significant difference was observed in the 1.5% group (*p* > 0.05). The 2.0% group exhibited the highest relative expression levels of the *cpt-1*, *lep*, and *srebp1* genes, which were significantly higher than those in the 0.5% group (*p* < 0.05), but no significant differences were observed in the 1.0% and 1.5% groups (*p* > 0.05). The 1.5% group exhibited the highest relative expression level of the *scd* gene, which was significantly higher than those of the 0.5%, 1.0%, and 2.0% groups (*p* < 0.05), as shown in Figure 2.

Compared to the control group (0% chitosan), dietary supplementation with chitosan at concentrations of 1.5% and 2.0% significantly up-regulated the relative expression levels of squalene epoxidase (*sqle*) gene in the liver of juvenile GIFT exposed to cadmium-induced stress (*p* < 0.05). The 1.5% group exhibited the highest relative expression level of the *sqle* gene, which was significantly higher than that of the 0.5% and 1.0% groups (*p* < 0.05), but no significant difference was observed in the 2.0% group (*p* > 0.05). However, the relative expression levels of the *sqle* gene in the 0.5% and 1.0% groups did not exhibit significant differences compared to that in the control group (*p* > 0.05), as shown in Figure 2.

## 4. Discussion

The present study demonstrated that, compared to the control group, dietary supplementation with chitosan significantly increased the contents of crude protein and crude fat in the muscles of juvenile GIFT exposed to cadmium-induced stress. These findings suggest that dietary supplementation with chitosan could exert a regulatory influence on muscle composition. The possible reasons could be attributed to the following factors. First, the presence of cadmium in the aquatic environment induced oxidative stress in fish through the generation of reactive oxygen species (ROS), and the fish’s physiological response involved an increased production of anions, hydroxyl radicals, and hydrogen peroxide. As a result, the fish expended significant energy on nutrient metabolism and ROS elimination within its system [21,22]. Second, due to the influence of cadmium-induced stress, the structural and functional integrity of biomacromolecules and various tissues and organs in fish were subjected to varying degrees of oxidative damage, leading to a reduction in nutrient absorption capacity and utilization rate as well as a decline in the fat and protein content of the fish [23,24]. Third, chitosan, a naturally occurring cationic polymer, demonstrated the capacity to chelate with cadmium in fish, thereby ameliorating its toxic effects. Moreover, chitosan exhibited an ability to sequester anions and scavenge hydroxyl free radicals, hydrogen peroxide, and other ROS, forming flocculates that alleviate oxidative stress-induced harm [25]. Fourth, the fish dietary supplementation with chitosan had the potential to enhance hepatic RNA repair, bolster protein synthesis, and regulate nutrient absorption and utilization in fish [26,27]. Fifth, chitosan exhibited the potential to augment the enzymatic activity involved in lipid and protein metabolism, thereby facilitating the proficient synthesis of lipids and proteins [28,29]. The dietary supplementation with chitosan resulted in a significant enhancement of crude protein content in yellow catfish (*Pelteobagrus fulvidraco*), as demonstrated by Li et al.’s study [30]. Likewise, Gheytasi et al. examined the impact of chitosan supplementation on rainbow trout (*Oncorhynchus mykiss*) and found notable enhancements in body composition, specifically an elevation in crude protein content relative to the control group [31].

The activities of lipase, trypsin, and amylase in the intestine can serve as indicators of the digestive efficiency of fish to a certain extent [32]. In this study, compared to the control group, the dietary supplementation with chitosan significantly increased the activities of lipase, trypsin, and amylase in the intestines of juvenile GIFT exposed to cadmium-induced stress. These findings suggested that dietary supplementation with chitosan exhibited potential for mitigating the adverse effects of cadmium stress on intestinal enzyme activity and improving digestive performance. The possible reasons could be attributed to the following factors. First, the accumulation of cadmium in fish leads to an increase in the abundance of harmful microbiota, such as spirochaetes and other pathogenic bacteria which colonize the intestinal tract of fish and induce diseases. Consequently, this resulted in a reduction in the proportion of beneficial bacteria within the fish gut microbiota, leading to the adhesion, damage, and cellular swelling of intestinal tissues. Therefore, this disrupted the activity and composition of digestive enzymes within the intestines [33,34]. Second, dietary supplementation with chitosan has been shown to augment the proliferation of beneficial intestinal bacteria, such as bifidobacteria and lactic acid bacteria, thereby modulating the composition of the gut microbiota. Consequently, this leads to enhanced intestinal metabolism, improved decomposition of toxic products under cadmium stress, heightened activity of digestive enzymes in the intestine, and increased digestive capacity for food [35,36]. Third, the chitosan molecule possesses a rich array of hydroxyl, acetyl, amino, and other functional groups, facilitating its adsorption onto the intestinal surface to establish a high-molecular-weight barrier film that effectively impeded the absorption of toxic substances by the intestine [35,37]. Fourth, chitosan possessed the capacity to induce the synthesis of anti-inflammatory compounds within the intestinal tract, thereby facilitating prompt restoration of intestinal damage and promoting recovery of food digestion and absorption capabilities. Additionally, the chemical constituents of chitosan contributed to fortifying the gut barrier function in fish while stimulating the secretion of beneficial substances and enhancing the production of digestive enzymes by intestinal cells [38,39,40]. Liu et al. administered chitosan-coated microfeed to large yellow croaker (*Larimichthys crocea*), resulting in a significant improvement in the intestinal digestive performance of the fish after 30 days [41]. Similarly, Asmaa et al. observed enhanced digestive performance in Nile tilapia (*Oreochromis niloticus*) through dietary supplementation with chitosan [36].

Heat shock protein (*hsp*) is a class of stress-inducible protein that is synthesized by organisms in response to adverse environmental stimuli [42]. Sonic hedgehog (*shh*) signaling molecules play a crucial role in regulating cellular development, and this pathway can activate tissue self-repair mechanisms [43]. Cytochrome P450 1A (*cyp1a*) serves as the primary activating enzyme involved in cell growth and development [44]. Metallothionein (*mt*) is an inducible non-enzymatic protein that exists within organisms [45]. The present study demonstrated that, compared to the control group, the dietary supplementation with chitosan could significantly up-regulate the relative expression levels of *hsp*, *shh*, *mt*, and *cyp1a* genes in the liver of juvenile GIFT exposed to cadmium-induced stress. These findings suggest that dietary supplementation with chitosan could effectively improve the stress resistance of GIFT. The possible reasons could be attributed to the following factors. First, cadmium exposure compromised the antioxidant and immune systems of fish, leading to inflammation [46]. Concurrently, heavy-metal stress disrupted the activity of antioxidant enzymes in fish by increasing ROS, resulting in oxidative stress, tissue, and organ damage, as well as diminished stress resistance [47]. Second, the chitosan molecular chain possessed numerous active groups with lone pair electrons, which could effectively coordinate with cadmium ions through coordination bonds. Consequently, a stable chelating polymer was formed, thereby ameliorating the toxicity of heavy-metal cadmium in fish [48,49,50]. Third, the expression of the *shh* signaling pathway was intricately linked to inflammation in vivo. Chitosan exhibited the capability to activate the *shh* signaling pathway in fish, thereby eliciting immune system stimulation for self-repair [51]. Fourth, the activation of the transcription factor NF-κB by chitosan occurred within the cytoplasmic milieu of damaged tissues, where it selectively bound to specific nuclear binding sites. This intricate interaction orchestrated the precise regulation of stress-resistance genes, thereby facilitating the proficient repair and restoration of compromised tissues and organs under cadmium-induced stress [52,53]. Fifth, exposure to cadmium stress resulted in an elevation of heavy-metal ion concentration within the body, leading to the induction of *mt* production. Chitosan facilitates an expedited formation of *mt*, which is complexed with cadmium ions which enables their non-toxic excretion from the system. This reduction in cadmium content mitigated adverse effects caused by cadmium stress on fish [54,55]. In the study by Brol et al., it was observed that the expression levels of *cyp1a* and glutathione peroxidase genes were significantly up-regulated in juvenile Pacific white shrimp (*Litopenaeus vannamei*) following chitosan administration to alleviate salt stress. This enhancement of defense mechanism and stress resistance under the dietary supplementation with chitosan conditions suggested potential benefits for juvenile Pacific white shrimp [56]. Sea cucumber (*Apostichopus japonicas*) was fed a dietary supplementation with chitosan by Wang et al., and after 8 weeks, it was observed that the expression of *hsp* significantly increased, resulting in enhanced stress resistance in the sea cucumber [57].

The regulation of lipid metabolism genes on the synthesis of enzymes involved in fat metabolism facilitates both lipogenesis and lipolysis, thereby playing a pivotal role in maintaining the homeostasis of lipid metabolism [58]. The present study demonstrated that compared to the control group, dietary supplementation with chitosan could significantly up-regulate the relative expression levels of hormone-sensitive lipase (*hsl*), peroxisome proliferator-activated receptor alpha (*pparα*), lipoprotein lipase (*lpl*), malate dehydrogenase (*mdh*), carnitine palmitoyltransferase 1 (*cpt-1*), leptin (*lep*), fatty acid synthase (*fas*), stearoyl-CoA desaturase (*scd*), peroxisome proliferator-activated receptor gamma (*pparγ*), and sterol regulatory element-binding protein 1 (*srebp1*) genes in the liver of juvenile GIFT exposed to cadmium-induced stress. These findings suggested that dietary supplementation with chitosan could effectively alleviate lipid metabolism impairment in juvenile GIFT exposed to cadmium stress, while concurrently augmenting their lipid metabolism capacity under such conditions. The possible reasons could be attributed to the following factors. First, the exposure to cadmium stress resulted in tissue and organ damage, disturbances in lipid metabolism, as well as suppression of the expression of genes associated with lipid metabolism in juvenile GIFT [59]. Second, the presence of chitosan enhanced the activity of digestive enzymes in the intestine, thereby facilitating nutrient absorption and promoting lipid metabolism [60]. Undoubtedly, the antibacterial and anti-inflammatory properties inherent in chitosan molecules significantly contributed to the preservation of intestinal and hepatic tissue integrity, thereby facilitating lipid metabolism within the organism [61]. Third, the chitosan–fat complex formed by chitosan in the digestive tract synergistically interacted with fatty acids and other substances, thereby augmenting the intestinal absorption of bile acids, facilitating the repair of fish intestinal damage induced by cadmium stress, and promoting the digestion and absorption of accumulated lipids in the fish intestine [62,63]. Fourth, the activation of chitosan on *ppar* could effectively suppress the NF-κB signaling pathway, thereby attenuating systemic inflammation, and facilitating in vivo fatty acid synthesis [64,65]. Fifth, the expression of *pparα*, *pparγ*, *lpl*, *scd*, and *fas* genes exhibited a decline at a chitosan concentration of 2.0%, potentially attributed to the fish’s inherent susceptibility, inadequate absorption, and utilization of high chitosan levels by the digestive system, and deposition of chitosan in the body leading to digestive burden and adverse effects, thereby impeding further promotion of lipid metabolism [66]. Similar investigations have been conducted, such as Liu et al.’s study on large yellow croakers raised with chitosan-coated microfeed, which up-regulated the expression of lipid metabolism genes in the fish [41].

## 5. Conclusions

In conclusion, dietary chitosan supplementation could alleviate the effects of Cd^2+^ stress on the muscle composition, digestive enzymes, lipid metabolism, and stress resistance, and their related gene expression, of juvenile tilapia, and to some extent reduce the toxic response of tilapia to Cd^2+^ stress. These results can provide scientific insights for the application of chitosan in aquaculture practices and the management of heavy-metal pollution in aquatic environments.

## Figures and Tables

**Table 1:** Composition of the experimental diets for juvenile GIFT (g/100g of dried feed).

Ingredients	Chitosan Levels (%)				
	0	0.5	1.0	1.5	2.0
Chitosan	0.00	0.50	1.00	1.50	2.00
Soybean oil	2.00	2.00	2.00	2.00	2.00
Fish oil	2.00	2.00	2.00	2.00	2.00
Fish meal	8.00	8.00	8.00	8.00	8.00
Rapeseed meal	22.00	22.00	22.00	22.00	22.00
Soybean meal	33.00	33.00	33.00	33.00	33.00
Dextrin	24.39	23.89	23.39	22.89	22.39
Gelatin	5.00	5.00	5.00	5.00	5.00
Vitamins mixture ^1^	1.00	1.00	1.00	1.00	1.00
Minerals mixture ^2^	1.00	1.00	1.00	1.00	1.00
Choline chloride	0.50	0.50	0.50	0.50	0.50
Sodium chloride	0.50	0.50	0.50	0.50	0.50
Adhesive ^3^	0.50	0.50	0.50	0.50	0.50
Attractant ^4^	0.01	0.01	0.01	0.01	0.01
Preservative ^5^	0.10	0.10	0.10	0.10	0.10
Proximate composition (%)					
Crude protein	33.88	33.88	33.88	33.88	33.88
Crude fat	7.35	7.35	7.35	7.35	7.35
Ash	6.96	6.96	6.96	6.96	6.96
Moisture	9.36	9.36	9.36	9.36	9.36
Crude fiber	5.56	5.56	5.56	5.56	5.56
Gross energy (Mcal/kg)	3.83	3.83	3.83	3.83	3.83

Note: ^1^ Vitamin mixture (IU or mg/kg of dried feed): vitamin A 2500 IU; vitamin D_3_ 1200 IU; vitamin K_3_ 60 IU; folic acid 5 mg; vitamin B_1_ 10 mg; vitamin B_2_ 10 mg; vitamin B_6_ 20 mg; vitamin B_12_ 0.15 mg; niacin 40 mg; calcium pantothenate 20 mg; inositol 150 mg; Biotin 0.2 mg; vitamin C 150 mg; vitamin E 60 mg. ^2^ Minerals mixture (mg/kg of dried feed): iron 15 mg; zinc 20 mg; manganese 2 mg; copper 1 mg; iodine 0.2 mg; selenium 0.05 mg; cobalt 0.25 mg; magnesium 0.06 mg; potassium 40 mg. ^3^ Adhesive: α-starch. ^4^ Attractant: nucleotides, betaine, amino acids, and taurine. ^5^ Preservative: sodium benzoate.

**Table 2:** Forward and reverse primers of detected genes for RT-qPCR.

Gene	Primer Sequence (5′→3′)	Amplicon Size (bp)	Gene Bank
*β-actin* ^1^	F: TGACCCAGATCATGTTTGAGACC	146	XM_031811226.1
	R: CTCGTAGATGGGTACTGTGTGGG		
*shh* ^2^	F: GGGAGAGGCAGACTGTAGAGATAGC	125	XM_003439222.5
	R: GACAAGCAGATGAGACCGACCAAC		
*hsp* ^3^	F: CAAGGTGATTTCAGACGGAGGGAAG	123	XM_003442456.5
	R: GCCTCTGCGATCTCCTTCATCTTC		
*mt* ^4^	F: AACGCCAGCATCACTCGGAAC	84	YP_003587621.1
	R: GCGGCAGGAACACTCACTCTTG		
*cyp1a* ^5^	F: AGAGTCAGTAGGCACAGTGTCCATC	129	NM_001279489.1
	R: GGGGCAAGTTGTTCCGATCAGAG		
*cpt-1* ^6^	F: ATTGGCAGGACAGCGACTACATTG	143	XM_019362661.2
	R: GGAAGGAGGTGAAGGGTCATCTAGG		
*pparα* ^7^	F: GTGGCTGCTATTATCTGCTGTGGAG	140	XM_019346353.2
	R: CTGGGGAAAAGGAAGGTGTCATCTG		
*hsl* ^8^	F: CAAGCGGCATCAGTCAGGAATAGG	80	XM_005463937.4
	R: CTCAACTCGGGGTCAATGGCATAC		
*lpl* ^9^	F: CTTCAGCCAGAACCAGCAGAGC	142	NM_001279753.1
	R: GTCGGTGGTGATGAGGAAGGATTG		
*mdh* ^10^	F: GGTGCTCGCTTCTTGTGGACAG	121	XM_005450070.4
	R: GACGGCCTCATTCTCATCTTCTTCC		
*lep* ^11^	F: GAAGTGGATCGCTGAGCATCTGG	129	XM_005449522.4
	R: CCATCCAAGCAGACCGTGACTATG		
*pparγ* ^12^	F: GTACACGGAGGCTACACGGAAAC	139	XM_019358463.2
	R: CTGCTTCTGCTGAACGAGACTGAC		
*fas* ^13^	F: AAGCCTTGTGTGCCTTCATCCAG	133	XM_003454056.5
	R: TCCCTGTGAGCGGAGGTGATTAG		
*srebp1* ^14^	F: GAACAGCAGCCGACAGATCACTC	116	XM_005473610.4
	R: TACAGCAGCCATTAACGAGCAAGTC		
*sqle* ^15^	F: CTGACGGGAGGAGGGATGAGTG	82	XM_003453510.5
	R: CATACAGGTCGGGAATGCTCTTGAG		
*scd* ^16^	F: ACAAGCTCTCCGTGCTGGTCAT	102	XM_005471382.2
	R: GCAGAGTTGGGACGAAGTAGGC		

Note: F: forward primer. R: reverse primer. ^1^ *β-actin*: internal reference gene; ^2^ *shh*: sonic hedgehog; ^3^ *hsp*: Heat shock protein; ^4^ *mt*: metallothionein; ^5^ *cyp1a*: cytochrome P450 1A; ^6^ *cpt-1*: carnitine palmitoyltransferase-1; ^7^ *pparα*: peroxisome proliferator-activated receptor alpha; ^8^ *hsl*: hormone-sensitive lipase; ^9^ *lpl*: lipoprotein lipase; ^10^ *mdh*: malate dehydrogenase; ^11^ *lep*: leptin; ^12^ *pparγ*: peroxisome proliferator-activated receptor gamma; ^13^ *fas*: fatty acid synthase; ^14^ *srebp1*: sterol regulatory element-binding protein 1; ^15^ *sqle*: squalene epoxidase; ^16^ *scd*: stearoyl-CoA desaturase.

**Table 3:** Effects of dietary supplementation with chitosan on the muscle composition of juvenile GIFT exposed to cadmium-induced stress (%/wet weight).

Index	Chitosan Levels (%)					*p*-Values
	0	0.5	1.0	1.5	2.0	
Moisture (%)	73.17 ± 1.23	72.67 ± 1.11	72.42 ± 1.37	72.48 ± 0.74	73.02 ± 0.63	0.401
Crude protein (%)	22.72 ± 0.25 ^b^	23.49 ± 0.30 ^a^	23.42 ± 0.37 ^a^	23.71 ± 0.43 ^a^	23.69 ± 0.23 ^a^	0.000
Crude fat (%)	6.97 ± 0.16 ^b^	7.38 ± 0.12 ^a^	7.43 ± 0.10 ^a^	7.58 ± 0.08 ^a^	7.50 ± 0.18 ^a^	0.000
Ash (%)	1.40 ± 0.02	1.40 ± 0.01	1.37 ± 0.01	1.37 ± 0.01	1.39 ± 0.01	0.297

Note: All above data are mean ± SE (*n* = 3 × 3 × 3), which means 3 parallel groups for each chitosan level, 3 fish samples for each parallel group, and 3 measured times for each fish sample. Different superscript letters in the same row indicate significant differences among the data (*p* < 0.05).

**Table 4:** Effects of dietary supplementation with chitosan on the intestinal digestive enzyme activities of juvenile GIFT exposed to cadmium-induced stress.

Index	Chitosan Levels (%)					*p*-Values
	0	0.5	1.0	1.5	2.0	
Lipase (U/mgprot) ^1^	0.12 ± 0.01 ^c^	0.27 ± 0.01 ^b^	0.28 ± 0.01 ^b^	0.33 ± 0.02 ^a^	0.32 ± 0.01 ^a^	0.000
Trypsin (U/mgprot) ^2^	8.21 ± 1.39 ^b^	18.31 ± 1.55 ^a^	18.58 ± 0.94 ^a^	19.24 ± 0.58 ^a^	18.24 ± 1.69 ^a^	0.000
α-amylase (U/mgprot) ^3^	1592.97 ± 100.05 ^b^	2764.49 ± 322.01 ^a^	2815.82 ± 185.95 ^a^	2912.66 ± 367.67 ^a^	2995.89 ± 249.55 ^a^	0.000

Note: ^1^ α-amylase unit definition: each milligram of protein in the tissue undergoes substrate interaction at a temperature of 37 °C for a duration of 30 min, and the hydrolysis of 10 mg starch is designated as one unit of α-amylase activity (U/mgprot). ^2^ Lipase unit definition: the reaction system operates at a temperature of 37 °C, where each gram of protein in the tissue undergoes substrate reaction for a duration of 1 min. The utilization of 1 μmol of substrate corresponds to one unit of lipase activity (U/mgprot). ^3^ Trypsin unit definition: the trypsin present in each milligram of protein in the tissue causes a change in absorbance of 0.003 per minute by a pH 8.0 and 37 °C environment, exhibiting one unit of trypsin activity (U/mgprot). All above data are mean ± SE (*n* = 3 × 3 × 3), which means 3 parallel groups for each chitosan level, 3 fish samples for each parallel group, and 3 measured times for each fish sample. Different superscript letters in the same row indicate significant differences among the data (*p* < 0.05).

## Data Availability

The original contributions presented in the study are included in the article, and further inquiries can be directed to the corresponding author(s).
